# Pannexins Are Potential New Players in the Regulation of Cerebral Homeostasis during Sleep-Wake Cycle

**DOI:** 10.3389/fncel.2017.00210

**Published:** 2017-07-17

**Authors:** Valery I. Shestopalov, Yuri Panchin, Olga S. Tarasova, Dina Gaynullina, Vladimir M. Kovalzon

**Affiliations:** ^1^Institute for Information Transmission Problems, Russian Academy of Sciences Moscow, Russia; ^2^Department of Ophthalmology, Bascom Palmer Eye Institute, University of Miami Miller School of Medicine Miami, FL, United States; ^3^Microbiology and Bioengineering Laboratory, Department of Genomics and Biotechnology, Vavilov Institute of General Genetics, Russian Academy of Sciences Moscow, Russia; ^4^Department of Mathematical Methods in Biology, Belozersky Institute, M.V. Lomonosov Moscow State University Moscow, Russia; ^5^Department of Human and Animal Physiology, Faculty of Biology, M.V. Lomonosov Moscow State University Moscow, Russia; ^6^State Research Center of the Russian Federation, Institute for Biomedical Problems, Russian Academy of Sciences Moscow, Russia; ^7^Department of Physiology, Russian National Research Medical University Moscow, Russia; ^8^Severtsov Institute Ecology and Evolution, Russian Academy of Sciences Moscow, Russia

**Keywords:** pannexins, non-REM sleep, purinergic system, cytokines, prostaglandin D2, glymphatic system, endothelium, neurovascular coupling

## Abstract

During brain homeostasis, both neurons and astroglia release ATP that is rapidly converted to adenosine in the extracellular space. Pannexin-1 (Panx1) hemichannels represent a major conduit of non-vesicular ATP release from brain cells. Previous studies have shown that Panx1^−/−^ mice possess severe disruption of the sleep-wake cycle. Here, we review experimental data supporting the involvement of pannexins (Panx) in the coordination of fundamental sleep-associated brain processes, such as neuronal activity and regulation of cerebrovascular tone. Panx1 hemichannels are likely implicated in the regulation of the sleep-wake cycle via an indirect effect of released ATP on adenosine receptors and through interaction with other somnogens, such as IL-1β, TNFα and prostaglandin D2. In addition to the recently established role of Panx1 in the regulation of endothelium-dependent arterial dilation, similar signaling pathways are the major cellular component of neurovascular coupling. The new discovered role of Panx in sleep regulation may have broad implications in coordinating neuronal activity and homeostatic housekeeping processes during the sleep-wake cycle.

## Introduction

Pannexins are a family of proteins that were discovered as a result of human and invertebrate gene homology analysis (Panchin et al., 2000). The members of this family, especially pannexin-1 (Panx1), is implicated in a number of vital biological functions and the development of several pathological mechanisms (Orellana et al., 2012; Prochnow et al., 2012; Penuela et al., 2013; Velasquez and Eugenin, 2014; Dalkara and Alarcon-Martinez, 2015). Although mouse Panx1-knockout (Panx1KO) models demonstrate a rather mild phenotype with no obvious alteration, as reported initially (Bargiotas et al., 2012; Dvoriantchikova et al., 2012), an essential physiological role of Panx1 was highlighted in a recent report of a human patient with a homozygous missense Panx1 variant (c.650G→A). This loss-of-function mutation resulted in extensive multisystem dysfunctions (Shao et al., 2016).

Panx1 is highly expressed in the brain (Bruzzone et al., 2003; Baranova et al., 2004), particularly in neurons (Ray et al., 2005; Zoidl et al., 2007), microglia and astrocytes (Huang et al., 2007), as well as in the cerebral vasculature (Burns et al., 2012). Its expression in the hypothalamus suggests a role in regulation of fundamental homeostatic processes (Ray et al., 2005; Hodson et al., 2015). This has been confirmed in a recent study highlighting an essential role of Panx1 in the regulation of the sleep-wake cycle (Kovalzon et al., 2017). Although not characterized mechanistically, this new function of Panx1 is attributed to its intimate integration with the brain purinergic system that plays a central role in regulation of sleep-wake cycle (Dunwiddie and Masino, 2001; Blanco-Centurion et al., 2006; Krueger et al., 2010; Blutstein and Haydon, 2013; Huang et al., 2014; Lazarus and Urade, 2015; Petit and Magistretti, 2016).

In the homeostatic brain, astrocytes are the main source of extracellular purines such as ATP and adenosine (Halassa et al., 2009; Bazargani and Attwell, 2016; Clasadonte et al., 2016). The mammalian purinergic system possesses two mechanisms for release of intracellular ATP: a transmembrane channel-mediated release and vesicular release (Lohman and Isakson, 2014; Burnstock, 2017). It is currently established that, along with some connexins, Panx1 hemichannels are the major conduit of non-vesicular release of intracellular ATP into the extracellular medium (Suadicani et al., 2012; Beckel et al., 2014). The non-vesicular release is responsible for ATP secretion in a circadian manner from astrocytes (Marpegan et al., 2011). Astrocytes are the main source of extracellular purines in the CNS (Halassa et al., 2009; Bazargani and Attwell, 2016; Clasadonte et al., 2016). Consistently, a recent report showed a role of Panx1 hemichannels in glucocorticoid-regulated diurnal oscillations of ATP release in spinal astrocytes (Koyanagi et al., 2016). To conclude, the central role of Panx in ATP secretion likely underlies Panx1 contribution to purinergic regulation of sleep-wake cycle.

In this article, we sought to survey different cellular and molecular mechanisms potentially connecting Panx1 hemichannels to regulation of the sleep-wake cycle. At present, it is clear from the literature that in addition to neuronal manifestations, natural sleep also engages with other physiological processes, such as cerebral blood circulation, brain ionic homeostasis and glymphatic clearance (Klingelhöfer et al., 1995; Braun et al., 1997; Xie et al., 2013; Jessen et al., 2015; Plog et al., 2015). Here, we review the well-known functional relationship between these processes and Panx and hypothesize on potential new ones.

## Pannexins in Neuronal Manifestations of Sleep

Despite recent progress in the understanding of the neural mechanisms of sleep regulation, the molecular nature of the “need for sleep” that gradually increases during wakefulness remains uncharacterized. However, we do know that adenosine is the principle endogenous somnogen in the brain milieu (Dunwiddie and Masino, 2001; Krueger et al., 2011, 2016; Porkka-Heiskanen and Kalinchuk, 2011; Tupone et al., 2013; Huang et al., 2014; Lazarus and Urade, 2015). In the anterior hypothalamus, daily fluctuations in adenosine concentration are associated with the sleep-wake cycle (Dunwiddie and Masino, 2001; Blanco-Centurion et al., 2006). Conversely, blockade of adenosine signaling through genetic ablation of A_1_ and A_2A_ receptors disrupts the sleep-wake cycle (Wei et al., 2011; Lazarus and Urade, 2015). A_2_ receptors are involved in the inhibition of brain stem activation system via enhancement of sleep-promoting GABA/galaninergic neurons in the ventrolateral preoptic area (VLPO; Obal and Krueger, 2003). In addition, cholinergic wake-promoting neurons in this brain area are inhibited through A_1_ receptors (Obal and Krueger, 2003).

Panx1 channels are likely involved in the regulation of the sleep-wake cycle via an indirect effect of released ATP on adenosine receptors. In a recent study, Kovalzon et al. (2017) revealed that Panx1KO mice have significantly prolonged periods of activity and spend less time in non-rapid eye movement (NREM) sleep, particularly during the dark (active) period compared with control C57Bl/6 mice. The effects of Panx1 knockout were similar to the influence of adenosine receptor antagonists such as caffeine and theophylline (Lazarus and Urade, 2015), which links NREM sleep alterations in Panx1KO mice to downregulation of adenosine signaling. In addition to the shorter NREM sleep, Panx1KO mice demonstrated a significant increase in movement activity. This is not surprising, because the blockade of adenosine signaling in the brain can dramatically alter behavioral patterns via effects on synaptic plasticity and learning (Ledent et al., 1997; Dunwiddie and Masino, 2001; Prochnow et al., 2012).

Along with that, sleep rebound after a 6-h sleep deprivation during the daylight remained unchanged in Panx1KO mice as compared to the control animals (Kovalzon et al., 2017). Presumably, the influence of Panx1 on homeostatic sleep following sleep deprivation can be masked by adenosine contributed by the pathways distinct from Panx1-mediated release of ATP. Indeed, our research showed that the release of ATP from the Panx1KO astrocytes is significantly reduced but not eliminated, as compared to WT mice (Beckel et al., 2014). The residual release can result in accumulation of adenosine during sleep deprivation, sufficient to induce a somnogenic effect in KO mice. Such explanation is supported by the experiments with a different mouse model, the ENT1 knockout mice, also possessing a decrease in cerebral adenosine level. These mice were also shown to have the lower baseline level of NREM sleep as well as the lack of any changes in sleep rebound (Kim et al., 2015). Therefore, the decreased basal level of adenosine does not necessarily translate into altered response to sleep deprivation.

In addition to the role in adenosine signaling, Panx may be involved in sleep/wake alterations that are mediated by some prostaglandins (PGs) and cytokines. Along with adenosine, PGD2, TNFα and IL-1β are among the most potent sleep-inducing substances (Fredholm, 2011; Krueger et al., 2011; Jewett and Krueger, 2012). Endogenous IL-1β and TNFα cytokines are expressed in various brain regions and different cell types, including neurons, microglia and astrocytes (Breder et al., 1993; Inoue et al., 2000; Vitkovic et al., 2000). Neurons release these factors in response to injury, ischemia and danger factors in a Panx1-dependent manner (de Rivero Vaccari et al., 2008, 2014; Abulafia et al., 2009; Dvoriantchikova et al., 2012). PGD2 and IL-1β signaling pathways are tightly linked to purinergic signaling through the inflammasome complex (Lutz et al., 2013; Cauwels et al., 2014; Meng et al., 2014; Zhang et al., 2015).

**Figure 1:**
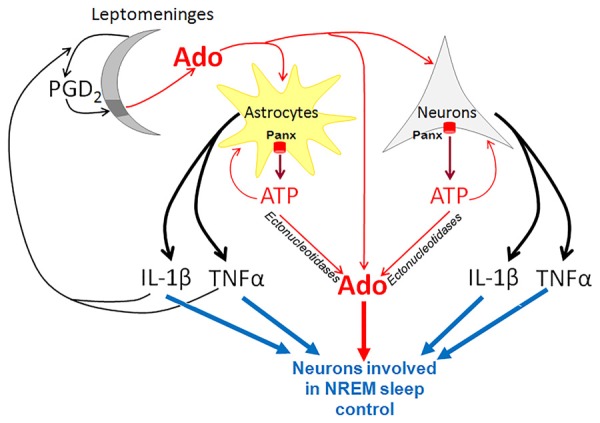
Pannexins (Panx) in purinergic control of non-rapid eye movement (NREM) sleep. In neurons and astrocytes, Panx proteins form plasma membrane hemichannels that are highly permeable for ATP. ATP is rapidly converted by ectonucleotidases to adenosine (Ado), which inhibits wake-active neurons and promotes the activity of sleep-active neurons in the basal forebrain. ATP also activates glial and neuronal P2 receptors and stimulates production of potent somnogenic substances such as TNFα and IL-1β. In addition, leptomeninges produce prostaglandin D2 (PGD2) that binds to its receptors solely in the meningeal area underlying the basal forebrain (dark gray). This binding results in elevation of Ado concentration in the basal forebrain parenchyma. TNFα and IL-1β derived from astrocytes and neurons stimulate the synthesis of PGD2.

Unrelated to inflammation, PGD2, TNFα and IL-1β are considered as fundamental players in the physiological regulation of normal sleep through a link to purinergic signaling (Figure 1; Hayaishi, 2000, 2011; Krueger et al., 2011, 2016; Urade and Hayaishi, 2011; Jewett and Krueger, 2012). Circulating cytokines may influence brain homeostasis by passing through the blood brain barrier either in the circumventricular organs or by activating receptors on the capillary endothelium, where they induce a secondary release of PGs and other signaling molecules (Konsman et al., 2002; Krueger et al., 2011). IL-1β and TNFα are upregulated in the brain during prolonged wakefulness, and the injection of these cytokines increases NREM sleep. Consistently, knockout mice lacking TNFα or IL-1β receptors spend less time in NREM sleep (Baracchi and Opp, 2008; Krueger et al., 2011, 2016; Jewett and Krueger, 2012).

The somnogenic activity of cytokines, such as IL-1β and TNFα, is linked to PGD2 production. IL-1β-induced NREM sleep is suppressed by inhibitors of cyclooxygenase, the key enzyme of PG production, while activation of the DP1/DP2 receptors increases the production of IL-1β and TNFα (Urade and Hayaishi, 2011). Interestingly, PGD2 in the brain is produced in the leptomeninges, rather than in the brain parenchyma (Hayaishi, 2000, 2011; Urade and Hayaishi, 2011). PGD2 is secreted from leptomeninges into the cerebrospinal fluid, and circulates throughout the ventricular and subarachnoidal spaces. The receptors for PGD2 are localized in a small area of leptomeninges on the ventrorostral surface of the basal forebrain just ventral to the VLPO.

The key role of Panx1 in ATP release during neuronal and glial transmission links this plasma membrane hemichannel to cytokine release and implicates it in sleep regulation via this pathway. Glial cells upregulate and release IL-1β and TNFα in response to extracellular ATP that binds to P2 receptors (Bianco et al., 2005; Krueger et al., 2010; Verderio and Matteoli, 2011). The somnogenic effect of PGD2 is also tightly linked to purinergic signaling, since it increases the extracellular level of adenosine in the brain. Adenosine is proposed to be the key signaling molecule that mediates the somnogenic effect of PGD2 (Mizoguchi et al., 2001; Hayaishi, 2011; Urade and Hayaishi, 2011; Lazarus and Urade, 2015). Consistent with this, the somnogenic effect of PGD2 infusion is attenuated by genetic ablation of A_2A_-receptors (Zhang et al., 2017). Activation of PGD receptors is followed by an increase in adenosine, which activates sleep-promoting neurons in the VLPO, and at the same time, down-regulates the histaminergic wake-promoting neurons in the tuberomammillary nuclei of posterior hypothalamus (Hayaishi, 2000, 2011; Urade and Hayaishi, 2011). These effects are abolished in DP receptor knockout mice.

Therefore, Panx1 hemichannels are likely implicated in the regulation of the sleep-wake cycle via ATP release. ATP is converted to somnogenic adenosine and both purines interact with other somnogens, such as IL-1β, TNFα and PGD2.

## Are Pannexins Involved in Regulation of the Glymphatic System?

The novel role of brain glia and our current understanding of the functional significance of sleep has been recently highlighted by the group led by Maiken Nedergaard (Xie et al., 2013; Jessen et al., 2015; Plog et al., 2015). They pioneered the discovery and characterization of the glymphatic (glial-lymphatic) system, a novel pathway of metabolic waste clearance in the brain. The activity of the glymphatic system during NREM sleep causes significant changes in the volume and ionic composition of the extracellular fluid in the brain (Iliff et al., 2012; Xie et al., 2013; Bedussi et al., 2015; Ding et al., 2016). The signal for glymphatic system activation is likely defined by the fall of noradrenergic tone that occurs at the onset of NREM sleep (O’Donnell et al., 2015). Fully activated during NREM sleep, the glymphatic system provides removal of potentially toxic waste and metabolites accumulated during prolonged wakefulness.

Considering that the astrocytic purinergic signaling system is closely linked to the regulation of the sleep-wake cycle (Blanco-Centurion et al., 2006; Halassa et al., 2009; Lazarus and Urade, 2015; Bazargani and Attwell, 2016; Clasadonte et al., 2016), we hypothesize that pannexin hemichannels may constitute an important functional component of the glymphatic system. Importantly, pannexin hemichannels were recently shown to be involved in transporting lactate across the astrocytic membrane (Karagiannis et al., 2016). Remarkably, rapid and sustained decline of lactate concentration marks NREM sleep (Naylor et al., 2012) and removal of lactate from the sleeping brain constitutes one of the key functions of glymphatic transport (Lundgaard et al., 2017).

## Pannexins in Control of Cerebrovascular Tone

Cerebral blood flow varies during the sleep-wake cycle. In comparison with wakefulness, total cerebral blood flow during periods of NREM sleep is significantly reduced (Klingelhöfer et al., 1995; Braun et al., 1997), but blood distribution within certain brain regions can vary significantly (Braun et al., 1997). Such cyclic changes in cerebral blood flow are associated with specific alterations in cerebrovascular tone, which may be regulated by the pannexin/purinergic system. Panx are widely expressed in the vascular bed and represent an essential pathway for the release of vasoactive purines (Billaud et al., 2012; Burns et al., 2012; Lohman et al., 2012a; Begandt et al., 2017). ATP and adenosine can dilate cerebral arteries and arterioles by activating endothelial P2 (P2Y1, P2Y2, P2Y4, P2Y6 and P2X4 subtypes and A_2a/b_ receptors, respectively; Ralevic and Dunn, 2015; Burnstock, 2017). Remarkably, endothelium sensitivity to ATP during the sleep-wake cycle is most pronounced at the beginning of the active phase (Durgan et al., 2016). Sleep disorders disrupt such cyclic variations of the dilator response to ATP (Durgan et al., 2016) and reduce its magnitude (Crossland et al., 2013).

In the endothelium, the Panx1 channel restrains vasoconstriction by regulating the secretion of vasorelaxing molecules by several proposed mechanisms. First, Panx1 activation could underlie endothelium sensitivity to shear fluid stress through their functional link to the PIEZO1 cation channel, a putative mechanosensor of endothelial cells (EC; Wang et al., 2016). This is followed by ATP release, eNOS phosphorylation, and production of nitric oxide (NO), a powerful vasodilator. Panx1 also mediates the eNOS-stimulating effects of calcitonin gene-related peptide (CGRP), a transmitter of sensory nerves (Gaete et al., 2014), which densely innervate pial arteries and arterioles (Hamel, 2006). Furthermore, Panx1 hemichannels may be co-activated with NMDA receptors by glutamate (Sandilos and Bayliss, 2012; Weilinger et al., 2012), a principal central nervous system excitatory transmitter that can induce NO-dependent relaxation of cerebral arteries (LeMaistre et al., 2012). Along with that, high levels of NO can act as a negative feedback loop to inhibit Panx1 hemichannels through the conventional cyclic GMP—protein kinase G pathway (Poornima et al., 2015) or by S-nitrosylation of Panx1 cysteine residues (Lohman et al., 2012b).

Besides modulation of NO-dependent signaling pathways, Panx participate in an endothelium-derived hyperpolarization (EDH) mechanism, which is independent of NO and PG (Edwards et al., 2010). Arteries of Panx1KO mice demonstrate an impaired endothelium-dependent relaxation (Gaynullina et al., 2014) due to the shortage of EDH (Gaynullina et al., 2015). In wild type mice, the EDH-component is inhibited by apyrase and an adenosine receptor antagonist, indicating the involvement of Panx1-mediated ATP release from EC. Extracellular ATP may provide an additional rise of cytoplasmic Ca^2+^ in EC through P2 receptors (Burnstock, 2017). Besides that, adenosine produced by ectonucleotidases, which are abundantly expressed in vascular cells (Kauffenstein et al., 2010; Zukowska et al., 2015) may activate the endothelium through A_2_ receptors (Burnstock, 2017). Functional deficiency of these mechanisms in Panx1KO mice supports Panx1 participation in endothelium-dependent control of vascular tone. Because a pannexin/ATP feed-forward mechanism can be activated by an increase in intracellular Ca^2+^ (Locovei et al., 2006b), this mechanism could underlie the generation of Ca^2+^ waves, that propagate along the endothelial cell layer and coordinate responses of smaller and larger resistance arteries (Locovei et al., 2006a).

Panx1 is also implicated as an important regulator of vasoconstriction. In smooth muscle cell (SMC)s of peripheral arteries Panx1 co-localizes with α_1D_-adrenergic receptors and secretes ATP that, in turn, activates P2Y-receptors and potentiates adrenergic vasoconstriction (Billaud et al., 2011, 2015). Importantly, Panx1-mediated vasoconstriction is specific to α_1D_-adrenergic stimulation and is lacking during smooth muscle stimulation with high-K^+^ depolarization, endothelin-1, or serotonin (Billaud et al., 2011, 2015). The latter observation questioned the role of this mechanism in cerebral vasoregulation, as cerebral arteries of different species, including humans, have demonstrated low reactivity to agonists of α-adrenoceptors (Högestätt and Andersson, 1984; Thorin et al., 2003; Bai et al., 2004).

**Figure 2:**
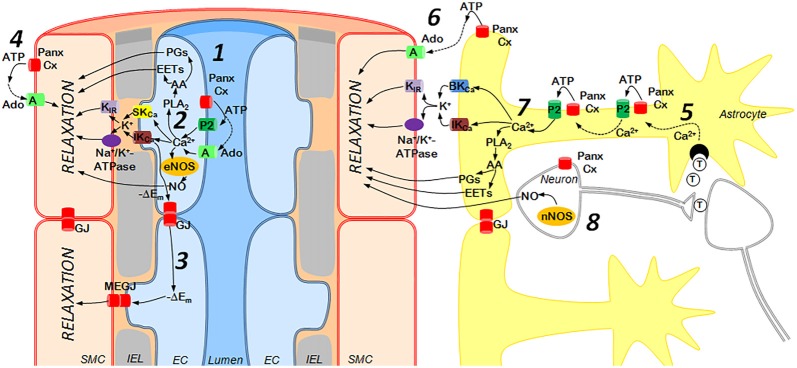
Purinergic mechanisms of cerebrovascular dilation: the role of endothelium (left) and astrocytes/neurons (right). *Left*: ATP is released from activated endothelial cells (EC) via Panx or Connexin (Cx) hemichannels and activates P2 receptors (subtypes P2Y1, P2Y2, P2Y4, P2Y6 and P2X4) or may be cleaved by ectonucleotidases to adenosine (Ado), an agonist of A receptors (subtypes A_2a_, A_2b_) (1). This elevates Ca^2+^ concentrations in EC and activates numerous mechanisms of vasorelaxation including (2): (i) SK_Ca_ and IK_Ca_ channel opening followed by the outward K^+^ current, activation of smooth muscle cell (SMC) K_IR_ channels and Na^+^/K^+^-ATPase and hyperpolarization of SMC (the endothelium-derived hyperpolarization (EDH) mechanism); (ii) phospholipase A2 (PLA_2_) activation, resulting in synthesis of vasodilators from arachidonic acid (AA); (iii) endothelial nitric oxide synthase (eNOS) activation followed by NO release. Hyperpolarization may spread along the endothelium via Panx or Cx gap junctions (GJ) and also may enter SMCs via myoendothelial gap junctions (MEGJ), formed by Panx or Cx (3). In addition, ATP released from SMCs may be cleaved to Ado, causing vasodilation via activation of A_2_ receptors (4). *Right*: Astrocytes in close proximity to interneuronal synapses can be activated by neurotransmitters (T), such as glutamate and ATP (5), leading to a rise in astrocytic Ca^2+^ concentrations, ATP release via Panx or Cx hemichannels, activation of P2 receptors and generation of Ca^2+^-waves (5). ATP released from astrocytes may be rapidly degraded to Ado, which relaxes SMCs through A receptors (6). In the astrocytic endfeet, increased Ca^2+^ concentrations may activate (7): (i) BK_Ca_ and IK_Ca_ channels followed by the outward K^+^ current, hyperpolarization (via K_IR_ and Na^+^/K^+^-ATPase) and relaxation of SMC; (ii) PLA_2_ resulting in synthesis of vasodilators from AA. In addition, neuronal NOS (nNOS) may produce NO (8). EC and astrocytes/neurons regulate cerebrovascular tone using similar mechanisms, such as K^+^ efflux through SK_Ca_/IK_Ca_ or BK_Ca_/IK_Ca_ channels followed by hyperpolarization and relaxation of arterial smooth muscle. Similarly, Prostaglandins (PGs) or epoxyeicosatrienoic acids (EETs) produced by either astrocytic or EC serve as vasodilators, as does NO derived from neurons or EC.

Small parenchymal arterioles possess a unique control mechanism, known as neurovascular coupling, that allows them to be regulated locally by surrounding neurons and astrocytes and provide adequate blood supply to active neurons (Figure 2). The mechanisms of neurovascular coupling are strongly dependent on purinergic signaling from neurons and astrocytes to vascular cells and pericytes (Pelligrino et al., 2011; Mishra et al., 2016; Mishra, 2017). While not fully established, a role of Panx in these processes is likely, as vasorelaxing factors derived from glial cells are similar to above-discussed factors of EC, and both cooperate with extracellular ATP (Vetri et al., 2011; Gaynullina et al., 2015; Longden et al., 2016; Mishra, 2017; Figure 2). Several lines of evidence support potential vasorelaxation mechanisms that involve Panx1. First, the signals from astrocytes and interneurons to vascular SMCs can be transmitted by such established endothelium-derived regulators as NO and arachidonic acid (AA) metabolites (Longden et al., 2016; Mishra et al., 2016; Mishra, 2017). Second, vasomotor signals from astrocytes involve an EDH-like mechanism with participation of Ca^2+^-activated K^+^ channels in astrocytic endfeet, K^+^ accumulation in local space between the feet and the SMC, and activation of K_IR_ channels and Na^+/^K^+^-ATPase in vascular SMCs with further hyperpolarization and relaxation (Longden et al., 2016). A moderate rise in extracellular K^+^ can activate pannexin channels (Suadicani et al., 2012; Wang et al., 2014) and may represent an additional pathway of Panx1-dependent regulation of cerebrovascular tone.

Potentially, purine/pannexin-dependent mechanisms of neurovascular coupling may vary in activity during the sleep-wake cycle. Thus, a somnogenic effect of glucose in mice is associated with an increase in adenosine concentration, and, consequently, dilation of parenchymal arterioles specifically in VLPO (Scharbarg et al., 2016). Moreover, the vasodilator effect of adenosine is dependent on the phase of sleep-wake cycle being significantly higher at the time of passive behavior in comparison to the active period. These data suggest an involvement of Panx in the control of sleep-wake cycle via the regulation of local blood flow.

## Conclusions

Recent observations suggest that Panx are involved in the regulation of the sleep-wake cycle. If experimentally validated, this will have broad implications in coordination of sleep-wake cycle-related changes in neuronal activity and homeostatic housekeeping processes, such as glymphatic clearance, regulation of cerebrovascular tone and many others. Therefore, various aspects of sleep-wake brain homeostasis including oscillating neuronal and glial activity and brain-vascular dynamics could all be linked by the same pannexin functions. In the near future, we expect that new evidence will be revealed that supports sleep-related functions of the pannexin/purinergic system that can potentially be used to design novel therapeutics to treat multiple types of sleep disorders.

## Author Contributions

VIS, YP, OST, DG and VMK contributed to the conception, literature review, writing and revising of this article.

## Conflict of Interest Statement

The authors declare that the research was conducted in the absence of any commercial or financial relationships that could be construed as a potential conflict of interest.

## Abbreviations

CGPR: calcitonin gene-related peptide
EDH: endothelium-derived hyperpolarization
EETs: epoxyeicosatrienoic acids
eNOS: endothelial nitric oxide synthase
NMDA: N-methyl-D-aspartate
NO: nitric oxide
NREM: non-rapid eye movement
Panx1: pannexin-1
Panx1KO: Panx1-knockout
PGs: prostaglandins
SK_Ca_, IK_Ca_ and BK_Ca_: calcium-activated potassium channels of small, intermediate and big conductance, respectively
VLPO: ventrolateral preoptic area.
